# Estimation of cardiac output in patients with congestive heart failure by analysis of right ventricular pressure waveforms

**DOI:** 10.1186/1475-925X-10-36

**Published:** 2011-05-13

**Authors:** Mustafa Karamanoglu, Tom Bennett, Marcus Ståhlberg, Vincent Splett, Barbro Kjellström, Cecilia Linde, Frieder Braunschweig

**Affiliations:** 1 NT&D Research, Medtronic Inc, Minneapolis, Minnesota, USA; 2 Department of Cardiology, Karolinska Institutet, Karolinska University Hospital, Stockholm, Sweden

**Keywords:** Ventricle, Pressure, Cardiac Output, Exercise, Pulmonary Artery

## Abstract

**Background:**

Cardiac output (CO) is an important determinant of the hemodynamic state in patients with congestive heart failure (CHF). We tested the hypothesis that CO can be estimated from the right ventricular (RV) pressure waveform in CHF patients using a pulse contour cardiac output algorithm that considers constant but patient specific RV outflow tract characteristic impedance.

**Method:**

In 12 patients with CHF, breath-by-breath Fick CO and RV pressure waveforms were recorded utilizing an implantable hemodynamic monitor during a bicycle exercise protocol. These data were analyzed retrospectively to assess changes in characteristic impedance of the RV outflow tract during exercise. Four patients that were implanted with an implantable cardiac defibrillator (ICD) implementing the algorithm were studied prospectively. During a two staged sub-maximal bicycle exercise test conducted at 4 and 16 weeks of implant, COs measured by direct Fick technique and estimated by the ICD were recorded and compared.

**Results:**

At rest the total pulmonary arterial resistance and the characteristic impedance were 675 ± 345 and 48 ± 18 dyn.s.cm^-5^, respectively. During sub-maximal exercise, the total pulmonary arterial resistance decreased (Δ 91 ± 159 dyn.s.cm^-5^, p < 0.05) but the characteristic impedance was unaffected (Δ 3 ± 9 dyn.s.cm^-5^, NS). The algorithm derived cardiac output estimates correlated with Fick CO (7.6 ± 2.5 L/min, R^2 ^= 0.92) with a limit of agreement of 1.7 L/min and tracked changes in Fick CO (R^2 ^= 0.73).

**Conclusions:**

The analysis of right ventricular pressure waveforms continuously recorded by an implantable hemodynamic monitor provides an estimate of CO and may prove useful in guiding treatment in patients with CHF.

## Introduction

Cardiac output (CO), together with measures of cardiac filling pressures and peripheral resistance, is a key variable to describe hemodynamic pathophysiology in patients with heart failure. At rest, heart failure patients often maintain a normal CO until later stages of the disease, when CO becomes too low to meet the metabolic demands of the body [1]. However, CO measurements during exercise reveal important information about the severity and prognosis of the disease [2,3] in patients with milder forms of congestive heart failure (CHF). Standard diagnostic tools to determine CO are commonly bound to the artificial laboratory environment and only provide situational information. Furthermore, the accurate assessment of CO usually requires invasive procedures that are associated with risks and costs. Therefore, continuous CO monitoring from an implanted sensor may overcome these obstacles and provide useful information to improve the management of patients with CHF.

CO can be affected by disorders that affect both left and right ventricular (RV) function [4,5]. CHF patients with elevated RV afterload have poor prognosis with a hazard ratio almost four times that of patients with normal RV afterload [5]. The true RV afterload, however, is not sufficiently reflected by simple mean pulmonary arterial pressure but is rather determined by a complex interaction of both steady (total pulmonary resistance) and oscillatory components (characteristic impedance and pressure wave reflection) [6] both of which are abnormal in CHF patients [7] at rest and during exercise [4].

Recently, continuous monitoring of right ventricular pressure parameters, recorded either from an implantable hemodynamic monitor (IHM) [8] or using a sensor incorporated in an implantable defibrillator [9], has been proposed to provide reliable long-term information on cardiac filling pressures in CHF patients. Using similar sensor technology, we have described a method to estimate CO from high fidelity RV pressure waveforms in an open chest canine model [10] and in humans with pulmonary arterial hypertension [11]. This algorithm assumes that the characteristic impedance of the RV outflow tract remains constant for a given individual [10] and accommodates the presence of pressure wave reflection [11].

The present study aims to investigate if this assumption remains valid in exercising heart failure patients and to assess if this algorithm can also be used to derive estimates of CO for continuous measurements in patients with heart failure who are implanted with an IHM or an ICD with pressure monitoring capabilities. For this purpose, retrospective hemodynamic data obtained during stationary bike tests were pooled with data from prospective chronic studies in patients implanted with an ICD implementing the algorithm to further validate these assumptions and to assess the bias and agreement of this method to the Fick method.

## Materials and methods

### Patient population and inclusion/exclusion criteria

Hemodynamic data from six male patients who were enrolled in a pilot trial [12] (Group I) and six male patients who were enrolled in the Chronicle^® ^Phase I technical feasibility study [13] (Group II) were retrospectively analyzed. Four male patients were then studied prospectively (Group III). In all groups, patients were included if they had chronic heart failure for >3 months in duration and were in NYHA-class II or III. Patients were excluded if they had any known uncorrected congenital heart disease, tricuspid or pulmonary valve stenosis, a mechanical right heart valve prosthesis, a previously implanted device in which transvenous leads remained in the heart, a terminal illness unrelated to their heart failure with life expectancy less than one year, were expected to undergo heart transplantation within 12 months or if they were unable to give consent. The institutional ethics committee approved the study and written, informed consent was obtained from each patient prior to enrollment.

### Study protocol

Patients in Group I and II received an IHM system (Chronicle^®^, Medtronic, Inc. Minneapolis, USA) which was implanted in the left subclavicular region using standard techniques for pacemaker insertion [14]. The RV lead, equipped with a pressure sensor was inserted via the subclavian vein or the internal jugular vein. The tip of the lead was positioned in the RV outflow tract and the pressure sensor was three centimeters proximal to the tip. Group III patients, implanted with a combined ICD and hemodynamic monitoring device implementing the algorithm for CO analysis (Chronicle-ICD, Medtronic, Inc. Minneapolis, USA) also received a standard bipolar defibrillation lead in the right ventricle [9].

Group I patients were studied at the time of the implant and performed submaximal bicycle exercise on an ergometer in supine position (6 minutes at 30 watts). Group II patients were studied at least one month after the implant and performed a two-staged upright bicycle exercise protocol (6 minutes at 25 watts followed by 3 minutes at 50 watts). Group III patients were studied at one and four months after implant at rest in supine position, standing and during upright bicycle exercise at 50 and 70% of the maximal work load. The maximal workload was determined during an exercise test one week before the one month test.

During exercise, pulmonary artery (PA) pressure, and mixed venous oxygen saturation were measured by an oximetric probe (Opticath, Oximetrix, Abbott Lab, Chicago, IL, USA). The ventricular pressure waveforms and arterial oxygen saturation were recorded by the IHM and by finger probe oximetry, respectively.

Oxygen consumption was measured breath-by-breath, using a metabolic assessment system (Groups I and II: CPX, Medgraphics, St Paul, MN, USA; Group III: Innocor, Innovision, Odense S, Denmark). For Groups I and II, breath-by-breath CO was calculated according to the Fick principle, using mixed venous oxygen saturation from the optical catheter, arterial oxygen saturation from the finger probe oximeter and oxygen consumption. For Group III, CO was calculated at the end of each intervention according to the Fick principle, using arterial and mixed venous blood samples and oxygen consumption. Beat-by-beat RV pressure waveforms, RV pressure derived estimated cardiac output (Group III) and breath-by-breath oxygen consumption values were saved to a computer disk for subsequent analysis.

### PCCO Algorithm

**Figure 1:**
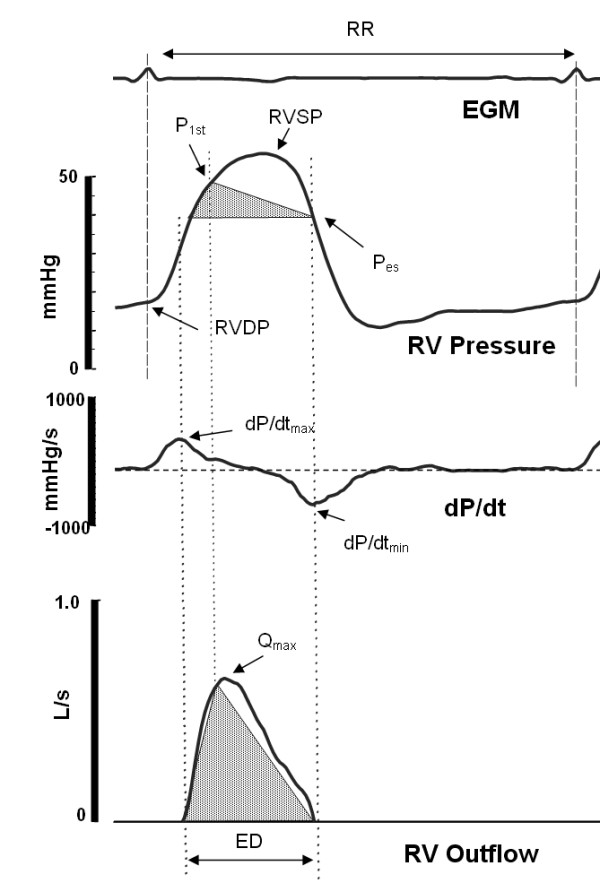
**A schematic illustration of the basic features of the RV pressure waveform (Top) and the identification of these feature points using the first derivative, dP/dt, of the RV pressure waveform (Middle)**. Three of these points identify the turning points of the RV outflow waveform approximated as a triangle (Bottom). The vertices of the flow waveform correspond to time of dP/dt_max_, peak flow (Q_max _)= time to early systolic shoulder of the RV pressure waveform (P_1st_), the time of dP/dt_min_. Hence, the triangle inscribed in the RV pressure waveform corresponds to the approximated RV outflow waveform. The ejection duration (ED) is defined as the difference between time of dP/dt_max _and time of dP/dt_min_, P_es _is the pressure at the time of dP/dt_min_. The RV diastolic pressure (RVDP) is measured at the time of the R-wave from the electrogram (EGM).

A beat-by-beat estimate of the CO was calculated using a previously established RV pressure waveform based pulse contour CO algorithm, PCCO [10,11]. This algorithm relies on the principle that the RV outflow contour can be identified in the RV pressure waveform using first and higher order pressure waveform derivatives [10, 11, 15-19, Figure 1). The algorithm provides an **estimate of the cardiac output**, eCO, as:(1)

where the ejection duration (ED) corresponds to the time interval between dP/dt_max _and dP/dt_min_. We have previously shown that the small error (<2%) introduced by triangular approximation of the outflow waveform can be ignored [10]. The P_1st _and P_ES _denote pressures at the time of peak flow and at the dP/dt_min_, respectively. The coefficient Z_c _is considered to be the patient specific RV outflow tract characteristic impedance unaffected by changes in pressure or flows. Note that if the value of Z_c _is unknown but assumed to be constant, Equation 1 denotes the cardiac output normalized to Z_c_, [10,11]. The value of Z_c _can easily be calculated empirically as the ratio of cardiac output (measured using a gold standard method) to first two terms of the Equation 1.

In Groups I and II, Equation 1 was implemented in dedicated software which was used to analyze the recorded RV pressure waveforms on a personal computer. In Group III, the PCCO algorithm was implemented in the hardware of the ICD which had the capability of recording the RV pressure waveform. Using this algorithm, beat-by-beat eCO values were calculated (Z_c _was assumed to be unknown) and stored in the ICD memory to allow measurements in chronic ambulatory settings. Using custom software, these stored eCO data were subsequently retrieved and analyzed.

### RV Afterload Parameters

Baseline and exercise values of steady and oscillator components of the RV afterload were calculated to assess differences between patients and influence of exercise. In particular, the changes in characteristic impedance due to exercise was calculated to verify the assumptions of the algorithm. Both the IHM and the ICD can analyze the RV pressure waveform on a beat-by-beat basis, extract and store the values of right ventricular diastolic pressure (RVDP), right ventricular systolic pressure (RVSP) and the estimated mean PA pressure (eMPAP) [12-15,20]. Using these hemodynamic parameters, the eCO and the directly measured cardiac output (mCO), the **outflow tract characteristic impedance**, Z_c_, was computed as the ratio of eCO to mCO and the **pulmonary arterial resistance **(PAR) was calculated as the ratio of eMPAP to mCO. Once PAR and the Z_c_, were known, the **coefficient of wave reflection**, Γ, was calculated as [6,15]:(2)

### Data Analysis and Statistics

The differences between baseline and exercise hemodynamic parameters were assessed using paired-t tests. Because the cardiac output measured by the breath-by-breath Fick technique (mCO) was highly variable (SD/mean = 35 ± 14%), measured and estimated parameters corresponding to rest and submaximal exercise were averaged over two minutes.

In Groups I and II, the effects of exercise on patient specific characteristic impedances, Z_c_, were analyzed and when it was found to be negligible, the average Z_c _for each patient, irrespective of the exercise stage was calculated using all beat-by-beat values. This average Z_c _was then entered in Equation 1 to calculate beat-by-beat eCO values.

In Group III, an average Z_c _for each patient, irrespective of the posture and exercise stage was calculated using data generated during the first exercise study at one month. This average Z_c _was then utilized to calculate beat-by-beat eCO values for the data collected at the four month exercise study using Equation 1.

A linear regression and Bland-Altman analyses were performed to assess the correlation coefficient, the mean bias and the limits of agreement of the eCO compared to mCO [21]. Finally, the tracking ability of the eCO to follow changes in mCO was assessed by calculating the eCO and mCO changes from their respective baselines and plotting these in a four quadrant and modified Bland-Altman plot along the lines described by [22]. Analyses were performed using Axum (ver 4.0, Mathsoft, Cambridge, MA). Data are expressed as the mean ± SD unless otherwise noted; p values < 0.05 were considered statistically significant.

## Results

Table 1 details the baseline characteristics of the study subjects. As a group, the patients had a Fick derived cardiac output of 4.9 ± 1.4 L/min at a heart rate of 77 ± 14 beats/min at rest. The RV diastolic, estimated mean pulmonary artery pressure and RV systolic pressures were above normal (RVDP: 13 ± 7 mmHg, eMPAP: 33 ± 12 mmHg, RVSP: 46 ± 19 mmHg) consistent with increased RV afterload. Both the steady (PAR: 675 ± 345 dyn.s.cm^-5^) and oscillatory components of the RV afterload (Z_c_: 48 ± 18 dyn.s.cm^-5^, Γ: 0.86 ± 0.04) were elevated compared to normal [7].

**Table 1:** The patient characteristics

	Patient No	Age (years)	**Body Surface Area (m**^**2**^**)**	Cardiac Output* (L/min)	NYHA Class	LVEF (%)	Aetiology
**Group I**	1	71	1.75	5.9	III	35	Ischemic
	2	67	2.42	4.1	III	17	Ischemic
	3	67	1.83	3.6	III	17	Ischemic
	4	60	1.75	3.3	III	20	Ischemic
	5	76	1.66	3.3	III	23	Ischemic
	6	79	1.81	4.8	II	40	Ischemic
							
**Group II**	7	70	2.01	5.8	III	25	Ischemic
	8	52	2.17	4.1	III	25	Ischemic
	9	61	2.23	6.0	III	20	Ischemic
	10	68	2.01	5.2	II	20	Ischemic
	11	63	2.19	4.3	II	30	Ischemic
	12	56	2.26	4.0	II	15	Ischemic
							
**Group III**	13	53	2.60	7.5	II	15	Idiopathic
	14	53	1.89	8.3	II	30	Ischemic
	15	66	2.00	4.1	II	30	Ischemic
	16	67	2.17	4.6	II	20	Ischemic
	**Mean ± SD**	**64 ± 8**	**2.01 ± 0.26**	**4.9 ± 1.4**	**2.1 ± 0.5**	**24 ± 7**	

* cardiac output measured at supine rest using breath-by-breath Fick (Groups I and II) and direct Fick (Group III) technique. NYHA = New York Heart Association, LVEF = Left Ventricular Ejection Fraction by echocardiography.

**Figure 2:**
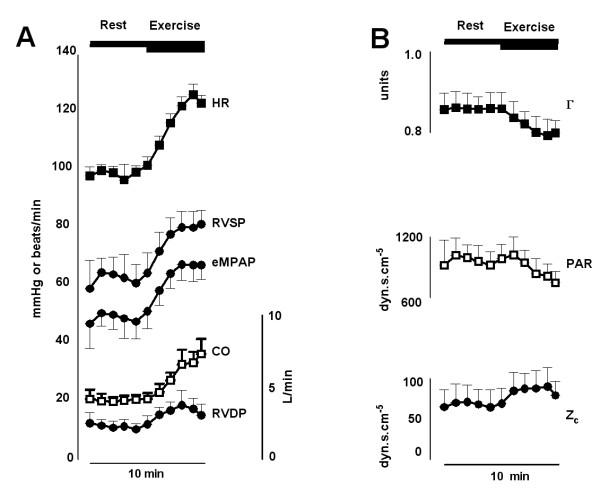
**(Panel A) The minute-by-minute changes in hemodynamic parameters (HR = Heart Rate, RVDP = Right ventricular end diastolic pressure, eMPAP = estimated mean pulmonary artery pressure, RVSP = right ventricular systolic pressure, CO = Cardiac Output) and (Panel B) the right ventricular afterload (Γ = coefficient of wave reflection, PAR = total pulmonary arterial resistance, Z**_**c**_**= characteristic impedance) parameters during the exercise protocol in one patient**. Bars correspond to periods during which rest and submaximal exercise data were averaged. Data are shown as mean and standard deviation for each one minute period.

**Figure 3:**
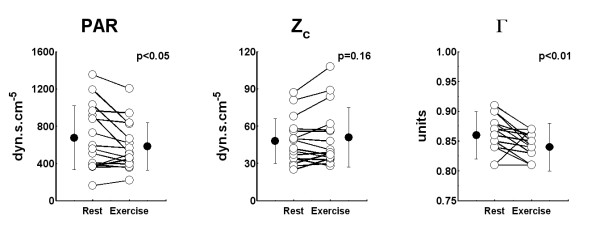
**The rest and submaximal exercise values of PAR = total pulmonary arterial resistance (Left), Z**_**c**_**= characteristic impedance (Middle) and Γ= coefficient of wave reflection (Right) in all 16 patients**. White circles = individual patients, black circles = group mean ± SD.

A representative tracing of the heart rate, RV pressures and the RV afterload parameters recoded during the exercise protocol is shown in Figure 2. Note that in this figure the error bars show the variability of the data during each one minute period in this patient. At sub-maximal exercise, both the pressures (RVDP: 22 ± 8 mmHg, eMPAP: 48 ± 10 mmHg, RVSP: 63 ± 15 mmHg) and the cardiac output (7.6 ± 2.5 L/min) were significantly increased compared to rest. This increase in cardiac output during exercise was due not only to an increased heart rate (77 ± 14 versus 101 ± 19 beats/min, p < 0.001) but also to an increased stroke volume (from 66 ± 21 to 77 ± 27 ml, p < 0.001). There was a significant decrease in PAR of -91 ± 159 dyn.s.cm^-5 ^and a significant decrease in the coefficient of wave reflection of -0.02 ± 0.04 units but no statistically significant change in Z_c _of 3 ± 9 dyn.s.cm^-5 ^during exercise (Figure 3). Since the effects of exercise on patient specific characteristic impedances, Zc, was found to be negligible, all beat-by-beat values of Z_c _obtained during the exercise test were averaged to estimate a mean Z_c _for each patient. This mean value was then used in Equation 1 to calculate beat-by-beat estimates of cardiac output.

**Figure 4:**
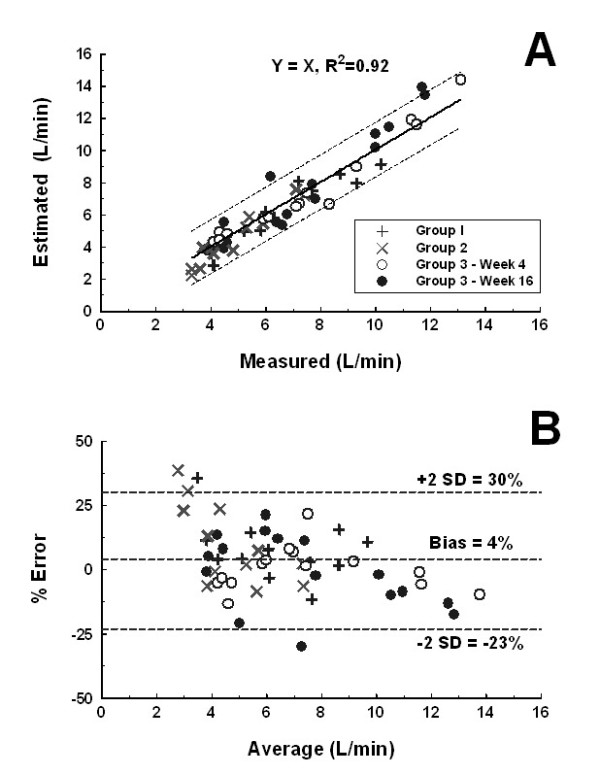
**(Panel A) Linear regression plot and (Panel B) Bland-Altman plot depicting the agreement between the measured and estimated cardiac outputs measured at rest and submaximal exercise**.

The eCO was similar to measured cardiac output, mCO, R^2 ^= 0.92, p < 0.001 (Figure 4, A). The Bland-Altman analysis indicated a mean bias of the estimate of 0.1 L/min (4%). The limits of agreement were -1.6 (-23%) to 1.7 L/min (30%) (Figure 4, B).

**Figure 5:**
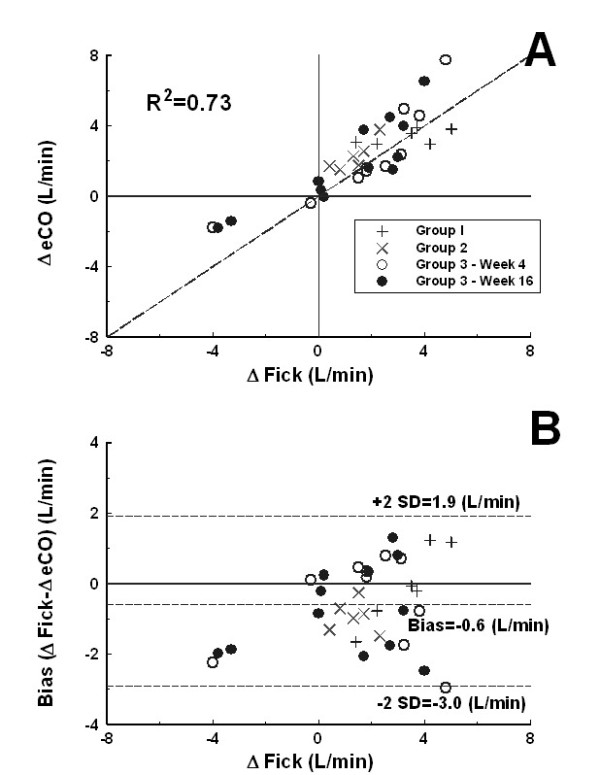
**The trend analysis using cardiac output change data from rest and exercise stages presented in four quadrants trend (Panel A) and modified Bland-Altman (Panel B) plots along the lines described by reference **[22]. Line of identity and the regression coefficient are given in panel A.

The trending ability of the algorithm using the four quadrant plot indicated that majority of the data points were close to the line of identity of the four quadrant plot (Figure 5, A). The trending effect was found to be significant (R^2 ^= 0.73). The corresponding modified Bland-Altman plot indicated a mean bias of the estimate of -0.6 L/min. The limits of agreement were -3.0 to 1.9 L/min (30%) (Figure 5, B) and there was no obvious sloping relationship between the two methods.

## Discussion

In this study, we used right ventricular pressure waveforms recorded with an implanted hemodynamic monitor, to estimate the cardiac output at rest and during bicycle exercise in patients with CHF. The results indicate that the PCCO algorithm and a single estimate of the characteristic impedance, individualized for each patient, are sufficient to provide estimates of the cardiac output at rest and during exercise. Given the important role of cardiac output in the hemodynamic pathophysiology of heart failure, continuous monitoring of this parameter over time using an implantable device may be helpful to improve the long-term management of CHF patients.

Critchley and Critchley suggested that in order to accept a new cardiac output measurement technique for clinical use, the limits of agreement should be within ± 30%[23], The present technique provides a range of -3.0 to 1.9 L/min which is within this recommended limit. In addition, serial measurements showed that changes in eCO tracked the changes in mCO (Figure 5A, R^2 ^= 0.73) [22]. It has been shown that the range of RV pressures monitored during daily living activities in patients with chronic heart failure is larger compared with a standard limited exercise test [24]. However, the conditions tested in our protocol likely represent the extremes of exertion and CO response experienced in the daily life of patients with chronic heart failure.

In this study, steady and oscillatory RV afterload parameters were measured. The results confirm that both the steady (the pulmonary arterial resistance, PAR), and oscillatory (the characteristic impedance of the RV outflow tract, Z_c_, and the coefficient of wave reflection, Γ) components of the RV afterload were already elevated in these patients with moderate HF [7]. The PCCO algorithm considers that the Z_c _of the RV outflow tract essentially remains constant for a given individual [10,11]. In this study, we found that during submaximal exercise the PAR decreased by ~17%, the amount of wave reflection was reduced by ~3% but the Z_c _of the RV outflow tract remained unchanged, confirming our initial hypothesis. Although the Z_c _appear to have quite large increase with exercise in some patients, this observation was not consistent in all patients (Figure 3, p = 0.16). This finding is consistent with earlier results, which suggested that the Z_c _of the pulmonary artery remains nearly constant in various conditions [7,25-27]. In the prospective arm of the study, we found that Z_c _again remained constant over 3 months as neither the error nor the ability to track was compromised (Figures 4 and 5). A theoretical study supported with empirical data is needed to better understand this interesting observation.

The present investigation extends previous reports on the performance of the PCCO algorithm from studies in acute open chest canines [10] and in humans with pulmonary arterial hypertension [11] to chronic heart failure patients. In the first study, we found that the PCCO algorithm provides good estimates of cardiac output during systematic preload, afterload and contractility changes [10]. In the second study, the PCCO algorithm given by Equation 1 yielded cardiac output estimates that were highly correlated with the breath-by-breath Fick cardiac output measurements during epoprostenol infusions in pulmonary hypertension patients [11].

The possibility for monitoring cardiac output has potential clinical implications in assessing and managing CHF patients. Strategies to determine and control hemodynamic status of patients with CHF in daily clinical practice commonly include regular clinic visits for the assessment of volume status and filling pressures by physical examination or various types of non-invasive measurements. However, the typical physical signs and symptoms of CHF have been shown to correlate poorly with measures of central hemodynamics [25] and non-invasive methods can be difficult to access. Moreover, since repeated cardiac catheterizations for estimation of cardiac output and RV afterload are associated with considerable risks, costs and inconvenience for the patient these measurements are generally performed at one or more discrete points in time without the disturbing influences of daily activities or stress.

To alleviate these shortcomings, an IHM which continuously records RV pressures and heart rate has been developed. Although still an investigational device, the value of IHM to predict deterioration in heart failure, preventing heart failure related hospitalizations and in tailoring drug treatment has been suggested in single and multicentre studies [28,29] and has been evaluated in large multicentre settings [8,9]. These developments would be further improved if the RV pressure waveforms recorded by the IHM could be utilized to estimate the cardiac output and the RV afterload. This information could enhance the long-term assessment of hemodynamics and the guidance of therapy in patients with heart failure without the need to perform invasive procedures. However, the accuracy and reliability of the PCCO algorithm over time still needs to be validated in a prospective study and the clinical usefulness of cardiac-output monitoring remains to be confirmed in clinical trials.

### Limitations

This study is limited by its small sample size and the retrospective application of the PCCO algorithm in a subset of the patients. The results obtained from the small group of prospective patients in Group III reflect findings to a period of 12 weeks and might require longer term studies for verification. Cardiac output was studied during supine and upright stationary bicycle exercise which may not reflect ambulatory conditions in patients performing daily living activities.

We considered that the level of error that the PCCO algorithm introduces is acceptable if one uses breath-by-breath or direct Fick method as the reference. It is possible that the PCCO algorithm might not be acceptable if a different reference, such as thermodilution, were to be used. In this study, we employed these Fick methods as reference because in the presence of tricuspid regurgitation, a potential when cathethers are introduced in the RV, the Fick reference was found to be superior to thermodilution [30].

It cannot be ruled out that the observed increase in cardiac output might also be due to reduced left ventricular afterload and improved left ventricular filling. Since in this study pulmonary arterial resistance, the steady component of RV afterload [6], was calculated any changes in LV filling pressures might confound our results.

In this study, we did not report and analyze the effect of some of the other hemodynamic parameters (pulmonary vascular resistance in Wood's Units, the resting and exercise blood pressures, etc.) and the medications (diuretics, ace inhibitors, beta blockers, etc.) on these findings. Further systematic studies with larger sample sizes are needed to address the influence of such parameters on these findings.

## Conclusions

In this study of patients with chronic heart failure, the RV pressure waveform recorded by an implantable hemodynamic monitor accurately and repeatedly measured cardiac output at rest and during exercise in an acute setting. This information, together with the corresponding data on cardiac filling pressures derived from the RV pressure sensor, could be useful in the long-term assessment of hemodynamic status and therapy response in heart failure patients.

## Competing interests

CL and FB have received research grants and been a consultant advisory and board member for Medtronic. MK, BK, VS and TB are employees of Medtronic, Inc.

## Authors' contributions

MK has conceived the original study, its design and coordination, contributed to the gathering and critical analysis of data and has written the manuscript. TB helped in the study design and coordination, and helped with the manuscript. MS has contributed to the cardiopulmonary exercise tests and to the interpretation of data. VS contributed to the gathering and the statistical analysis of data and the drafting of the manuscript. BK has contributed to the study design and coordination and cardiopulmonary exercise tests and the drafting of the manuscript. CL helped in recruiting the patients and coordination of the study and critical review of the manuscript. FB helped its design and coordination, performed catheterizations and contributed to the gathering and critical analysis of data and has helped write the manuscript. All authors read and approved the final version of the manuscript.
